# Behavior in motivational conflicts is determined by magnitude of potential outcomes and relates to anxiety levels

**DOI:** 10.21203/rs.3.rs-6916454/v1

**Published:** 2025-06-24

**Authors:** Shir Ben-Zvi Feldman, Omer Dayan, Ya’ira Somerville, Chang-Hao Kao, Shelly Cohen, Oriana Glickman, Sonia G. Ruiz, Philip Newsome, Julia O. Linke, Tomer Shechner, Daniel S. Pine, Silvia Lopez-Guzman, Rany Abend

**Affiliations:** 1. School of Psychology, Reichman University, Herzliya, Israel; 2. Unit on Computational Decision Neuroscience, National Institute of Mental Health, Bethesda, MD, USA; 3. Department of Psychology, Yale University, New Haven, CT, USA; 4. Department of Psychology, University of Southern California, Los Angeles, CA, USA; 5. Department of Psychology, Freiburg University, Freiburg im Breisgau, Germany; 6. School of Psychological Sciences and the Integrated Brain and Behavior Research Center, University of Haifa, Israel; 7. Section on Development and Affective Neuroscience, National Institute of Mental Health, Bethesda, MD, USA

**Keywords:** approach, avoidance, motivation, anxiety, conflict

## Abstract

Human behavior often involves resolving conflicts between motivations to pursue rewards and to avoid harm. Maladaptive resolution of such approach-avoidance conflicts is a hallmark of various psychopathologies, notably anxiety disorders. To systematically study motivated behavior tendencies, we need to identify factors that may drive them, such as sensitivity to the magnitudes of expected outcomes. We developed a novel paradigm that presents conflict situations with parametrically- varying magnitudes of potential monetary gains and losses that map onto a continuous behavioral outcome reflecting willingness to engage in each situation. Using this paradigm, we evaluate the hypothesis that potential outcome magnitudes determine conflict behavior, across a series of studies in different populations and settings - including a proof-of-concept with young adults, replication in a larger sample, online administration, and application to youth with and without anxiety disorders. Our findings demonstrate that outcome magnitudes reliably predicted behavior, yielding robust individual indices of gain-approach and loss-avoidance tendencies. Moreover, anxiety severity was associated with greater passive avoidance in a sample-specific manner. By quantifying individual-level indices that link potential outcome magnitudes to observable behavior, our work offers a reliable framework for investigating adaptive and maladaptive motivated behaviors, with potential utility for both basic and clinical research.

## Introduction

Behavior reflects motivations to approach desirable goals and avoid harmful ones [1–4], processes that are fundamental for survival across species [1, 5–11]. As energy demands increased among more complex species, expanded foraging introduced risks such as predation, often generating a conflict between approach and avoid motivations [1, 12]. Consequently, evolution favored the development of mechanisms enabling the representation of potential, competing outcomes, and the capacity to arbitrate among them such that the optimal behavior is executed [4, 13, 14].

Given that individuals exhibit different tendencies in seeking reward versus avoiding aversive stimuli, interindividual differences in motivational tendencies can have a significant impact on how both are weighed for the resolution of motivational conflict [15–16]. It is therefore unsurprising that disturbances in motivational behaviors and in the adaptive resolution of motivational conflicts are key features of major psychopathologies [17–21]. For example, major depression is associated with diminished approach motivation and behavior towards appetitive stimuli, and enhanced valuation of negatively valenced stimuli in the resolution of goal-related conflict [18–19, 22–23]. In contrast, substance use disorders are associated with heightened approach tendencies and a disproportionate pursuit of reward-related outcomes despite potential negative consequences [18–19, 24–25].

Aberrant patterns of motivated behaviors are especially prominent in anxiety disorders which are characterized by excessive and pervasive avoidance behavior [14, 17, 26–27]. Specifically, hallmark anxiety symptoms are excessive active avoidance behavior when faced with a concrete, increasingly imminent threatening object or situation, and excessive passive avoidance, reflecting diminished approach towards desired rewards within motivational conflicts due to perceived potential harm [17, 23, 28–32]. Passive avoidance is a particularly impairing aspect of pathological anxiety whereby individuals are less likely to pursue a wide range of potentially beneficial situations, such as professional advances and social interactions, due to overweighing potential danger (loss) relative to reward (gain) [17, 33–34]. Moreover, persistent passive avoidance may contribute to maintenance and further generalization of symptoms as it becomes habitual while preventing normative experiences of eventual safety [19, 33–37], and is especially difficult to address in treatment [33–34, 36–37].

The centrality of motivated behaviors in human life, and especially their maladaptive expression in psychopathology, raises the need for their quantification in experimental settings [38]. Building upon the foundational work of previous research [e.g., 5, 29, 39–43], our study introduces specific adaptations aimed at enhancing mechanistic understanding of motivated behaviors and resolution of conflicts. By integrating these adaptations, we seek to address the need for reliable and valid quantification of approach and avoid tendencies, thereby contributing to the ongoing advancement of this field.

There are several key methodological and conceptual challenges in the study of motivated behavior [33, 39–40, 44–45]. First, research on motivational conflicts often indexes approach and avoidance behaviors separately, or their net observable outcome behavior [29, 39–40, 46]. Concurrent quantification of both approach and avoidance tendencies, at the individual-subject level, could inform on their respective, independent contributions to the resolution of motivational conflict and observed behavior [28–29, 39, 41, 47]. For example, quantification of both motivations to approach potential rewards and actively retreat from potential punishment may disentangle passive and active avoidance tendencies [14].

Second, real-world scenarios of motivational conflicts naturally feature a wide range of potential gain and loss domains and magnitudes and accordingly elicit varying magnitudes of approach and avoid behaviors, respectively [14, 28–29, 39]. For example, different employment opportunities may offer varying degrees of monetary reward and thus elicit varying degrees of approach motivation; at the same time, they may also involve different levels of potential negative consequences, such as pressure or risk of failure, thereby evoking varying degrees of avoidance motivation. To accurately quantify individual differences in motivational tendencies, i.e., map potential outcomes onto behavior, paradigms may benefit from featuring a wide range of potential outcome magnitudes. Along these lines, considering that behavior is often not monolithic but rather reflects a continuous gradient of expression accommodating motivational factors (e.g., vigor and effort), paradigms that offer a continuous range of behavioral output may enable more complete outcome-behavior mapping [28–29, 39, 44, 48].

Finally, given the strong relevance of perturbations in motivated behaviors to psychopathology, paradigms that are appropriate for administration in target clinical populations may propel mechanistic and clinical research. For example, as noted, excessive and persistent passive avoidance is a hallmark feature of pathological anxiety, and appears to already be observed in youth [17, 49–52]. Thus, lab tasks designed to capture this impairing symptom [e.g., 51, 53] may potentially generate significant clinical value if designed to be child-appropriate.

While research has begun to address these challenges, further efforts are needed to develop and validate appropriate behavior assessment tools. To address some of these gaps, we introduce the Doors task, designed to quantify behavioral tendencies within motivational conflict. The task centers on magnitudes of potential gain and loss outcomes as determinants of behavior within motivational conflicts. To this end, it involves parametrically varying magnitudes of conflicting potential monetary gain and loss outcomes and requires participants to use a joystick to behaviorally express the extent of their willingness to engage in these different conflict scenarios. Larger potential gains are expected to lead to greater movement forward (greater willingness to engage in the situation), reflecting stronger gainapproach behavior tendencies; larger potential losses are expected to lead to greater movement away, reflecting stronger loss-avoidance behavior tendencies. This design models a wide range and complexity of motivational conflict while allowing for naturalistic approach/avoid movement along a continuous behavioral outcome scale from complete avoidance to complete approach. Through a multi-level analytic design, we then independently compute subject-level indices of gain-approach and loss-avoid tendencies, as well as conflict avoidance. Finally, the task was designed to be simple and engaging, facilitating its administration among target populations such as patients and children.

Here, we report on a series of four studies. The first study presents a proof of concept and evaluation of task effects in a sample of young adults, including assessment of reliability of derived behavior indices. In the second study, we assess the replicability of our initial findings in an independent sample. In the third study, we examine the generalizability of the task to a web-based version of the paradigm. In the fourth study, we further evaluate the generalizability of the task to pediatric anxiety patients and examine associations between indices and symptoms.

We hypothesized, first, that expressed behavior in the task would be determined by the magnitudes of potential outcomes, such that larger potential gains would generate greater approach behavior tendencies, while larger potential losses would lead to greater avoidance behavior tendencies; these tendencies are expected to be stable within individuals. Second, given theoretical and empirical links between anxiety and passive avoidance behavior, we hypothesized that greater anxiety severity would be associated with passive avoidance behavior. These same hypotheses were examined in each of the four studies, although we also anticipated some potential differences in outcomes arising from the different populations probed and differing modes of task delivery.

## Study 1: Initial Assessment of the Doors approach-avoid behavioral task

### Overview

This study conducted a preliminary, proof-of-concept examination of the Doors task to quantify magnitude-dependent motivational behavior tendencies within conflicts in a sample of undergraduate university students. To this end, task behavior indices reflecting gain-approach (*β_Gain_*), loss-avoid (*β_Loss_*), and conflict avoidance (*β_Conflict_*) tendencies, were calculated. In addition, we examined preliminary associations with anxiety and depression symptom severity.

### Results

**Fig. 1.**
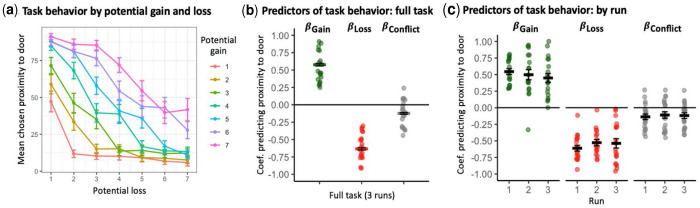
Study 1 - Proof-of-Concept Young Adult Sample: task behavior and derived predictors of behavior. (**a**) Impact of different magnitudes of potential loss and gain on task behavior (chosen proximity to the door), indicating a pattern of greater chosen proximity with greater potential gain and with smaller potential loss. (**b**) Distribution of derived motivational predictors of behavior (in terms of beta coefficients predicting chosen proximity based on outcome magnitude) for the gain (*β_Gain_*), loss (*β_Loss_*), and conflict (*β_Conflict_*) magnitudes, in the full task (across 3 runs). (**c**) Distribution of derived behavior predictors for the task runs (1-3). Horizontal crossbars reflect sample means; error bars reflect standard error of the mean.

All participants completed the task without any reported difficulty. Figure 1a depicts the mean chosen proximity to the door by magnitudes of potential gain and loss, indicating a pattern of decreasing proximity with decreasing potential gain and with increasing potential loss. The linear mixed-effects model indicated that potential gain magnitude, *β_Gain_*, was a significant and positive predictor of behavior (chosen proximity), *M*=0.58, *t*(2891)=31.35, *p*<0.001; see Fig. 1b and Supplement for more details. Similarly, potential loss magnitude, *β_Loss_*, was a significant and negative predictor of behavior (chosen proximity), *M*=−0.63, *t*(2891)=−34.37, *p*<0.001. These predictors were also significant at the individual-participant level, for all participants. Behavioral coefficients including model statistics are presented in Table S1 in the Supplement. Participant age and biological sex were not associated with chosen proximity to the door, *p*s>0.73.

*β_Gain_* and *β_Loss_* did not differ in absolute magnitude, *p*=0.17, and were significantly and negatively correlated, *r*(18)=−0.64, *p*=0.002 (see Table S2). This negative correlation indicates that participants who were more responsive to potential gains (more positive *β_Gain_*) tended to be more responsive to potential losses (more negative *β_Loss_*), suggesting a shared, general motivational tendency. Moreover, the magnitude of conflict, *β_Conflict_*, also predicted behavior, although in some individuals, greater conflict was associated with approach behavior tendencies (positive coefficients) and in others, avoidance behavior tendencies (negative coefficients); see Supplement. Behavior predictor coefficients did not covary with participant sex or age, *p*s>0.42.

To assess the reliability of behavior indices, we calculated predictor coefficients for each of the three runs separately (see Fig. 1c and Table S3), and their cross-run ICC values. The three behavior coefficients exhibited good reliability: *β_Gain_*: ICC=0.80, *F*(19,38)=5.10, *p*<0.001; *β_Loss_*: ICC=0.84, *F*(19,38)=6.10, *p*<0.001; and *β_Conflict_*: ICC=0.89, *F*(19,38)=9.50, *p*<0.001. Further, *β_Gain_*, *β_Loss_*, and *β_Conflict_* coefficient values did not significantly change across runs (paired-samples *t*-tests), *t*(19)s<1.30, *p*s>0.21.

In this sample, *β_Conflict_* correlated negatively with GAD-7, *r*(18)=−0.61, *p*=0.005 (see Table S4), and non-significantly but at trend-level with STAI, *r*(18)=- 0.40, *p*=0.08, suggesting that greater anxiety symptoms are associated with conflict avoidance. *β_Gain_* and *β_Loss_* did not significantly correlate with anxiety or depression severity, *p*s>0.18. See Supplement for additional effects.

### Conclusion

Consistent with our first hypothesis, findings from Study 1 indicate that larger magnitudes of potential gains and losses generate stronger approach and avoid behaviors, respectively. Individual-level indices of behavioral tendencies derived from the task were reliable across task runs. While the specific hypothesized link between anxiety and passive avoidance (our second hypothesis) did not emerge in this sample as expected via *β_Gain_* values, a related pattern was observed whereby greater anxiety symptoms were associated with greater conflict avoidance, reflecting a withdrawal from engaging in risky situations, regardless of absolute outcome magnitudes.

## Study 2: A replication and extension study of the Doors approach-avoid task

### Overview

The primary aim of this study was to replicate the initial behavioral findings from Study 1 *β_Gain_*, *β_Loss_*, and *β_Conflict_* reflecting magnitude-based predictors of behavior) in a larger undergraduate sample. Given participant feedback in Study 1, we modified the task to include only two runs. Additionally, we explored subjective perceptions of task performance via a series of questions answered following the task (see Methods).

### Results

**Fig. 2.**
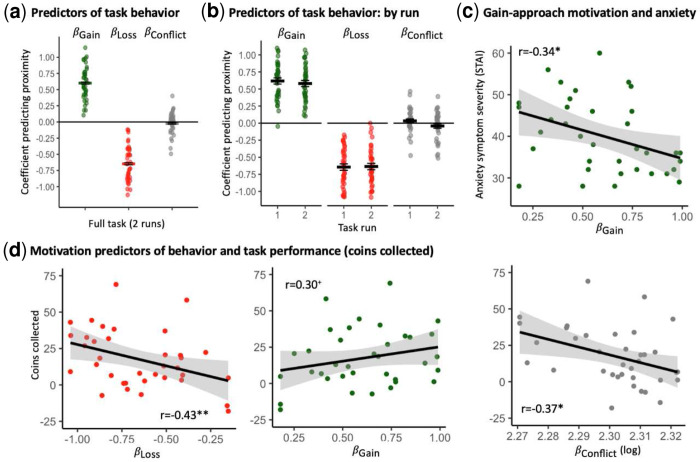
Study 2 - replication sample: Derived motivational predictors of behavior and associations with anxiety severity and task performance. (**a**) Distribution of derived motivational predictors of behavior (in terms of beta coefficients predicting chosen proximity based on outcome magnitude) for the gain (*β_Gain_*), loss (*β_Loss_*), and conflict (*β_Conflict_*) magnitudes, in the full task (across 2 runs). (**b**) Distribution of derived behavior predictors for the task runs (1–2). (**c**) A negative correlation between *β_Gain_* and anxiety levels (STAI), suggesting that greater anxiety is associated with reduced conflict-related approach behavior (passive avoidance). (**d**) Correlations between the number of coins collected and β values for loss, gain, and conflict. **, *p*<0.01; ***, *p*<0.001. Horizontal crossbars reflect samples means; error bars reflect standard error of the mean. In scatterplots, the black line reflects the regression line and the shaded area reflects the 95% confidence interval.

See Fig. S1 for descriptive depiction of task behavior. Study 2 results replicated those of Study 1, with potential gain magnitude (*β_Gain_*) and loss magnitude (*β_Loss_*) significantly predicting positive and negative chosen proximity to the door, respectively, *p*s<0.001; see Fig. 2a, Table S1, and Supplement for complete details. These predictors were all significant at the individual-participant level among all participants. Conflict magnitude, *β_Conflict_*, was likewise a predictor of behavior, with overall average conflict avoidance across the sample, *p*=0.023. As in Study 1, *β_Gain_* and *β_Gain_* were significantly and negatively correlated, *r*(35)=−0.82, *p*<0.001 (see Table S2). Behavior coefficients largely did not covary with participant sex or age, *p*s>0.20; there was a trend-level sex difference in *β_Conflict_*, with females showing stronger conflict avoidance relative to men, *F*(1,35)=4.04, *p*=0.052. Behavior coefficients for the two runs (see Fig. 2b and Table S3) exhibited good reliability, ICCs=0.76–0.84, and coefficient values for *β_Gain_* and *β_Gain_* did not significantly change across the two runs, while *β_Conflict_* values became more negative between runs 1 and 2 (see Supplement).

In terms of symptoms, *β_Gain_* correlated significantly and negatively with STAI scores (see Fig. 2c and Table S4), suggesting that greater anxiety is associated with diminished conflict-related approach behavior, i.e., passive avoidance. As in Study 1, *β_Conflict_* correlated negatively with anxiety severity; however, in this sample, this association was not significant, *p*=0.19; see Supplement for additional effects.

The larger sample in this study enabled us to examine additional task effects. Specifically, we calculated the mean number of collected coins across the task as a measure of task performance given the task goal. *β_Loss_* correlated significantly and negatively with coins collected, *r*(35)=−0.43, *p*=0.008, while *β_Gain_* correlated positively, but only at trend level, with coins collected, *r*(35)=0.30, *p*=0.071; see Fig. 2d and Table S5. These findings suggest that a stronger link between potential outcome magnitude and relevant behavior (more negative *β_Loss_* coefficients, more positive *β_Gain_* coefficients) is associated with better task performance. Additionally, *β_Conflict_* correlated significantly and negatively with coins collected, *r*(35)=−0.37, *p*=0.024, suggesting that greater conflict avoidance (negative *β_Gain_* coefficients) is associated with fewer collected coins.

**Fig. 3.**
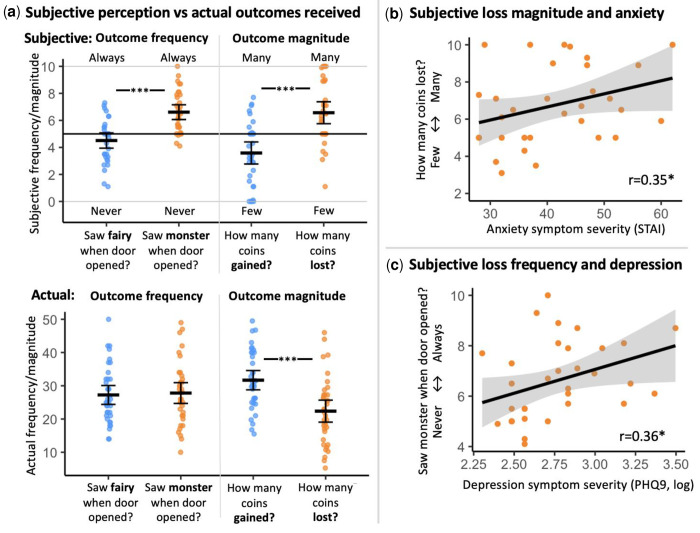
Study 2 - replication sample: Biases in subjective perception of experienced outcomes. (**a**) The top panel depicts participants’ post-task subjective perception of outcome frequency (how often received gains or losses, left) and magnitude (how many coins gained or lost, right), compared to actual delivered task outcomes depicted in the bottom panel. Across the sample, participants overestimate loss frequency and magnitude. (**b**) Participants’ perception of loss magnitude was positively correlated with anxiety symptom severity (STAI, trait subscale of StateTrait Anxiety Inventory). (**c**) Participants’ perception of loss frequency was positively correlated with depression symptom severity (PHQ-9, Patient Health Questionnaire-9; log-transformed). The black lines in Panels B and C indicate the linear regression line, with the shaded area representing the 95% confidence interval. *, *p*<0.05; ***, *p*<0.001. Horizontal crossbars reflect samples means; error bars reflect standard error of the mean. For display purposes, the number of collected coins was divided by 4. In scatterplots, the black line reflects the regression line and the shaded area reflects the 95% confidence interval.

Additionally, exploratory analyses of retrospective estimation of outcomes indicated a loss bias, with participants perceiving losses as more frequent and of greater magnitude than they actually were, while gains were underestimated. These subjective biases were related to individual differences in anxiety and depression symptoms. For full statistical details, see Supplementary Table S6 and Fig. 3.

### Conclusion

Findings from Study 2 replicate the capacity of the Doors task to reliably quantify behavior within motivational conflicts as a function of potential gain and loss magnitudes, consistent with our first hypothesis. While gain and loss magnitudes were strongly uniform in their direction of behavior prediction (e.g., all sample *β_Gain_* coefficients were positive), conflict magnitude demonstrated substantial inter-subject variability in yielding approach and avoid behaviors. Supporting our second hypothesis, greater anxiety severity was associated with diminished approach behavior within conflict, i.e., passive avoidance. Individuals with a stronger link between potential outcome magnitude and relevant behavior (more negative *β_Loss_* coefficients, more positive *β_Gain_* coefficients) demonstrated better performance per task goals (collected more coins); however, greater conflict avoidance was associated with fewer collected coins. Finally, participants demonstrated biases in retrospective assessment of their experience, overweighing loss frequency and magnitude; these biases were associated with anxiety and depressive symptom severity.

## Study 3: Generalization to a wider subject pool through an internet-based platform

### Overview

In this study, we aimed to replicate the behavioral findings from Studies 1 and 2 (prediction of behavior by *β_Gain_*, *β_Loss_*, and *β_Conflict_*) while extending the task in two major ways. First, we sought to generalize lab findings to a wider subject pool that goes beyond the narrow characteristics of undergraduate samples, particularly with regard to a broader age range. Second, given the increasing use of online platforms for delivering cognitive and behavioral tasks [54–55], we wanted to examine whether the task generates similar effects through such delivery medium. To this end, we translated the Doors lab task to a 3-run version that can be administered via the Prolific platform, which required using a mouse instead of a joystick for behavior.

### Results

**Fig. 4.**
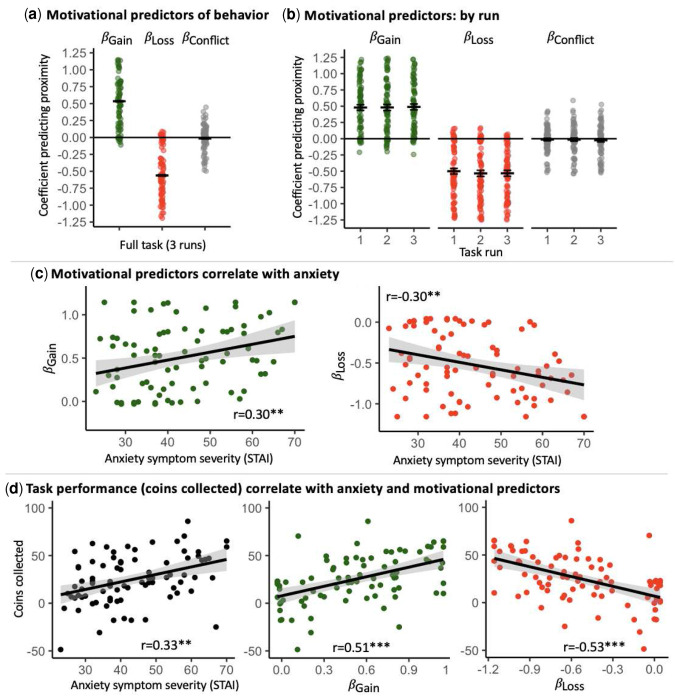
Study 3 - online sample: Derived motivational predictors of behavior and associations with anxiety and task performance. (**a**) Distribution of derived motivational predictors of behavior (in terms of beta coefficients predicting chosen proximity based on outcome magnitude) for the gain (*β_Gain_*), loss (*β_Loss_*), and conflict (*β_Conflict_*) magnitudes, across the task (3 runs). (**b**) Distribution of behavior predictors for each of the task runs (1–3). (**c**) Gain (*β_Gain_*) and loss (*β_Loss_*) motivational predictors correlate with anxiety symptom severity (State-Trait Anxiety Inventory scores). (**d**) Task performance (mean number of coins collected) correlates with anxiety symptom severity (left) and with gain (*β_Gain_*, center) and loss (*β_Loss_*, right) motivational predictors. **, *p*<0.01; ***, *p*<0.001. Horizontal crossbars reflect samples means; error bars reflect standard error of the mean. In scatterplots, the black line reflects the regression line and the shaded area reflects the 95% confidence interval.

See Fig. S2 for descriptive depiction of task behavior. Replicating the previous studies, the linear mixed-effects model indicated that *β_Gain_* and *β_Loss_* were positive and negative predictors of chosen door proximity, respectively, *p*s<0.001; see Fig. 4a, Table S1 and Supplement for more details. Additionally, in this sample, *β_Conflict_*_^_ was a significant and negative predictor of proximity, *p*=0.018. RT was significantly associated with chosen proximity, *M*=0.06, *t*(10666)=6.82, *p*<0.001, while sex and age were not, *p*s>0.17. As in the previous studies, *β_Gain_* and *β_Loss_* were significantly and negatively correlated, *p*<0.001 (see Table S2 for detailed statistics), while age and sex were not correlated with *β_Gain_*, *β_Loss_*, or *β_Conflict_*, *ps*>0.14. Behavior coefficients exhibited good reliability across the runs, ICCs=0.85–0.93, and *β_Gain_*, *β_Loss_* and *β_Conflict_* coefficients did not significantly change across runs, all *p*s>0.17; see Fig. 4b and Table S3, and Supplement.

In terms of associations with symptoms, as in Study 2, *β_Gain_* correlated significantly with anxiety severity (STAI scores); however, in this sample, the correlation was positive, *r*(82)=0.30, *p*=0.005; see Fig. 4c and Table S4, and Discussion. *β_Loss_* correlated negatively with anxiety severity, *r*(82)=−0.30, *p*=0.006, while *β_Conflict_* did not, *p*=0.75. A similar pattern of effects was noted for GAD-7 and PHQ-9 scores, see Supplement. Thus, in this sample, stronger motivational action was associated with symptoms of anxiety and depression. Given the wide age range in the sample, we calculated partial correlation coefficients, controlling for age, as well as sex, in the above analyses. These indicated that significant associations were age- and sex-invariant.

Task performance, quantified by mean number of coins collected, was positively associated with anxiety severity (STAI scores), *r*(82)=0.33, *p*=0.002, suggesting that more anxious participants retained more money; see Fig. 4d. A similar pattern of effects was noted for GAD-7 and PHQ-9 scores, see Supplement. The number of collected coins also correlated positively with *β_Gain_*, and negatively with *β_Loss_* (see Table S5).

**Fig. 5.**
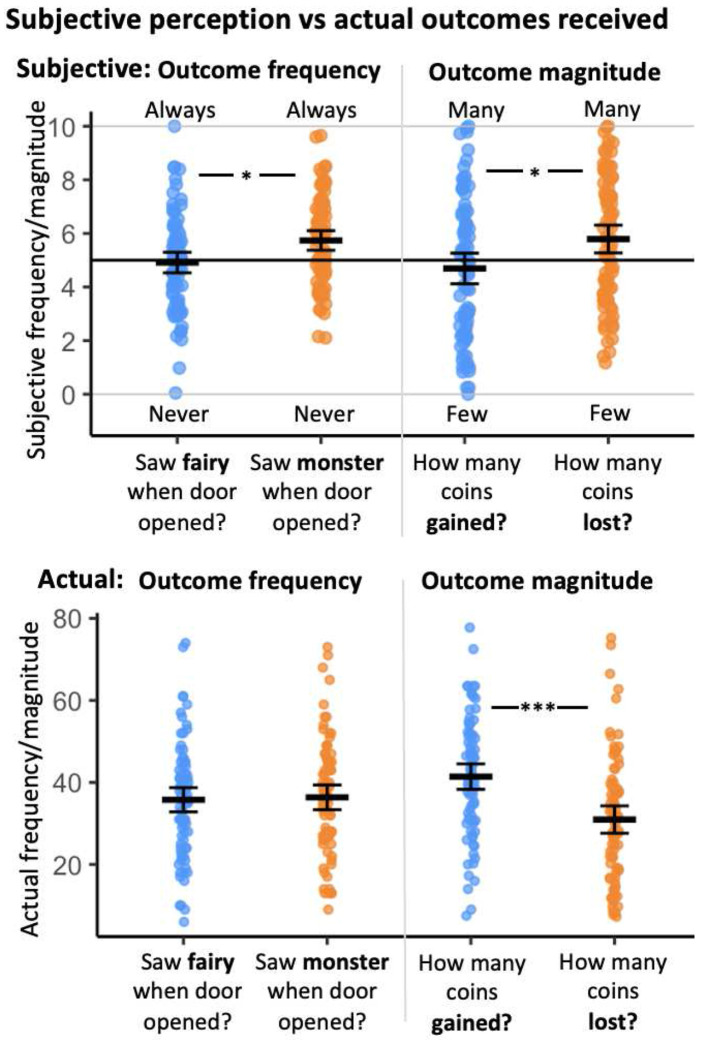
Study 3 – online sample: Biases in subjective perception of experienced outcomes. The top panel depicts participants’ post-task subjective perception of outcome frequency (how often received gains or losses, left) and magnitude (how many coins gained or lost, right), compared to actual delivered task outcomes depicted in the bottom panel. Across the sample, participants overestimate loss frequency and magnitude. *, *p*<0.05; ***, *p*<0.001. Horizontal crossbars reflect samples means; error bars reflect standard error of the mean. For display purposes, the number of collected coins was divided by 4.

Finally, we examined participants’ retrospective subjective ratings of outcome frequency and magnitude. Consistent with Study 2, results suggested a robust loss- related bias: participants tended to overestimate the frequency and magnitude of losses relative to gains, despite no objective difference in actual outcomes. These biases did not significantly correlate with symptom severity. (See Supplementary Table S6 and Fig. 5 for full statistical details).

### Conclusion

Consistent with our first hypothesis, findings from Study 3 replicate those of Studies 1 and 2, demonstrating reliable quantification of gain-approach and loss-avoid behavioral tendencies within motivational conflicts when the task is administered via an online platform. Similarly to the previous studies, participants with greater *β_Gain_* and smaller *β_Loss_* coefficient magnitudes collected more coins. Consistent with Study 2, participants demonstrated loss-related biases in subjective assessment of their experiences. Finally, as in the previous studies, anxiety severity correlated with motivational behavior tendencies; however, in contrast to our second hypothesis, in this online sample, anxiety (and depression) severity was positively associated with *β_Gain_* coefficient magnitude. Interestingly, anxiety severity was also correlated with *β_Gain_* , supporting the relationship between anxiety and active avoidance of negative outcomes. Together, this study indicates replicability of derived behavior indices and subjective biases but suggests that the nature of associations with symptoms may depend on the type of populations sampled or mode of delivery.

## Study 4: Extending the task to a clinical, pediatric population

### Overview

In patients with psychopathology, the suboptimal resolution of approach-avoid conflicts is strongly associated with maladaptive behavioral responses that constitute key symptomatology. Pathological anxiety is a particularly notable example, involving early-emerging, pervasive, and impairing excessive avoidance behavior. Following Studies 1–3, we conducted Study 4 to examine whether the task can capture differences in motivational tendencies between a sample of pediatric anxiety patients and healthy, age-matched controls. This enables us to examine the suitability of the task for youth populations as well as extend the previous findings on associations with anxiety symptoms to a clinical sample. Given the prior studies, we hypothesized that anxious relative to healthy youth would show excessive passive avoidance reflected in diminished gain-approach behavior as well as enhanced conflict avoidance.

### Results

See Fig. S3 for descriptive depiction of task behavior. In line with the previous studies, the linear mixed-effects model indicated that among youth as well, *β_Gain_* and *β_Loss_* positively and negatively predicted chosen door proximity, respectively, *p*s<0.001. In this sample, door proximity was predicted by *β_Conflict_* non-significantly and only at trend level (see Table S1), and was not predicted by RT, sex, age, or diagnostic group, *p*s>0.14. As before, *β_Gain_* and *β_Loss_* were significantly and negatively correlated, *p*<0.001 (see Table S2); *β_Loss_* and *β_Conflict_* were significantly and positively correlated, *r*(45)=0.32, *p*=0.028. Behavior coefficients did not covary with sex or age, *p*s>0.22. Coefficients exhibited moderate to good reliability, ICCs=0.71–086, and did not significantly change across runs; see Supplement. Thus, youths can provide reliable indices of motivated behavior in the task (See Table S3).

**Fig. 6.**
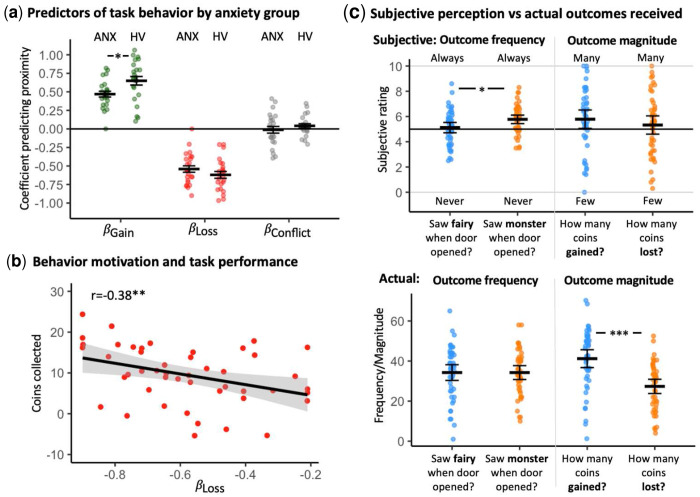
Study 4 - pediatric anxiety sample: Derived motivational predictors of behavior, associations with anxiety and task performance, and biases in outcome perception. (**a**) Distribution of derived motivational predictors of behavior (in terms of beta coefficients predicting chosen proximity based on outcome magnitude) for the gain (*β_Gain_*), loss (*β_Loss_*), and conflict (*β_Conflict_*) magnitudes for the anxiety patients (ANX) and for the healthy volunteers (HV) comparison group in the full task (across 3 runs). (**b**) Greater motivational tendency (in loss domain) is associated with better task performance (in terms of mean number of coins collected, log-transformed here). (**c**) The top panel depicts participants’ post-task subjective perception of outcome frequency (how often received gains or losses, left) and magnitude (how many coins gained or lost, right), compared to actual delivered task outcomes depicted in the bottom panel. Across the sample, participants overestimate loss frequency and magnitude. *, *p*<0.05; **, *p*<0.01; ***, *p*<0.001. Horizontal crossbars reflect samples means; error bars reflect standard error of the mean. In scatterplots, the black line reflects the regression line and the shaded area reflects the 95% confidence interval. For display purposes, the number of collected coins was divided by 4.

ANOVA of *β_Gain_* coefficients indicated significantly lower gain-approach behavior in the anxiety group relative to the healthy volunteer group, *F*(1,45)=6.83, *p*=0.012, in line with the anxiety-*β_Gain_* association observed in Study 2; see Fig. 6a and Table S4 for summary of anxiety associations across studies. No significant differences were noted for *β_Loss_*, and *β_Conflict_*, *p*s>0.22. At the dimensional level, *β_Gain_* coefficients correlated negatively with anxiety severity (SCARED scores), though only at trend level, *r*(45)=−0.26, *p*=0.072. As in Studies 2 and 3, *β_Loss_* was negatively associated with the mean number of accumulated coins *r*(45)=−0.38, *p*=0.009 (see Fig. 6b and Table S5), while *β_Gain_* was not, *p*=0.28.

As in prior studies, participants showed a subjective bias in evaluating outcome frequency and magnitude, overestimating losses relative to gains, despite no such objective differences. This pattern was not related to anxiety severity (see Supplementary Table S6 and Fig. 6c for full results).

### Conclusion

Consistent with our first hypothesis, findings from this pediatric sample demonstrate that outcome magnitudes within motivational conflicts predicts behavior in youth and across clinical status. Supporting our second hypothesis, a significant link was found between anxiety severity and passive avoidance (i.e., diminished gainapproach behavioral tendency) replicating the finding from Study 2 and suggesting a potentially meaningful association. However, it should be noted that a similar association emerged in Study 1 via conflict avoidance, and was inversed in Study 3, highlighting variability across samples. Additionally, as in the previous studies, participants with a larger *β_Loss_* coefficient magnitude collected more coins. Finally, youth exhibited similar loss-related biases in subjective assessment of their experiences to adults.

### Discussion

In this study, we introduce the Doors task, designed to test whether gain and loss potential outcomes systematically influence behavior within motivational conflicts. Quantification of subject-level, outcome magnitude-driven behavior tendencies was achieved by mapping parametrically varying magnitudes of potential gain and loss, presented within an ecological decision-making context, onto a continuous behavioral outcome. Indices of behavioral tendencies were then examined in different populations and modes of delivery.

Through four experiments, several findings emerged. First, gain-approach and loss-avoid behavioral tendencies emerged reliably and robustly across different sample populations and modes of delivery. Thus, in line with our first hypothesis, the magnitudes of potential gains and losses drive relevant behaviors and individual-level indices of these links can be quantified by the task. Second, magnitudes of motivated behavior tendencies related to real-world-compatible task performance (collecting task coins with monetary equivalence). Third, and partially consistent with our second hypothesis, in three samples, including treatment-seeking pediatric anxiety patients, greater anxiety severity was associated with stronger passive avoidance or conflict avoidance tendencies (although note that different anxiety measures were used across studies; see Table S4); in the online-delivery sample, an inverse, yet significant, pattern was noted. Finally, the task reliably elicited subjective biases in retrospective assessment of experienced losses; these biases showed inconsistent associations with anxiety and depressive symptom severity. Together, these findings identify outcome magnitudes as significant predictors of behavior within conflict and provide evidence for the Doors task’s potential utility in basic and clinical research on adaptive and maladaptive motivated behaviors.

#### Subject-level indices of motivated behaviors

Our primary finding was that across all samples, encompassing different settings and populations, participants exhibited sensitivity to magnitude of potential gain (β_Gain_) and loss (β_Loss_) as strong predictors of the extent of approach and avoid behaviors, respectively. Another measure derived from this task, *β_Conflict_*, reflecting sensitivity to motivational conflicts independently of gain-approach and loss-avoid tendencies [44], showed greater variability in its sign, with some participants demonstrating conflict approach behavior (positive coefficient values) and others showing conflict avoidance (negative coefficient values). These findings are in line with prior work linking binary behavioral choice preferences to potential outcome magnitude [28, 56–57] and neural responses in brain regions associated with behavior selection and execution [58]. Importantly, our work extends such findings by mapping parametrically varying outcomes magnitudes onto a continuous measure of behavior, allowing for greater range and sensitivity of individual differences in the expression behavioral tendencies. As such, our findings suggest that approach and avoidance behaviors should be considered along a continuous dimension of magnitude, rather than binary.

The moderate to good reliability of these latent indices of behavior tendencies indicate their utility for quantitative research on behavior in motivational conflicts. Importantly, good reliability was observed in an online version of the task (via the Prolific platform), suggesting the task lends itself to administration through platforms that enable massive data collection within short timeframe, offering considerable advantages to behavioral research [54–55]. Moreover, we show that the task likewise generates behavior indices with adequate reliability in children and adolescent populations, including among treatment-seeking patients. Given the centrality of aberrant motivational behavior in numerous psychopathologies [19, 59], as well as the emergence of pathological anxiety in young age [60], tools for quantification of behavioral tendencies in such population is critical for the advancement of clinical research [51].

#### Linking enhanced task performance with increased behavioral sensitivity to outcome magnitudes

An important finding from this study is the link between individual differences in behavioral tendencies and performance in the task as defined by its goal of accumulating as many task coins as possible (equivalent to real-world money). Such a goal may be seen as corresponding to life norms and expectations, especially in Western cultures [61–62], and as such task findings may inform on individuals’ motivational tendencies as these relate to what is considered as adaptative or maladaptive behavior. Specifically, we found that participants who exhibited a stronger linear link between potential gain and loss magnitudes and approach and avoid behaviors (i.e., more positive βGain coefficients and more negative βLoss coefficients), respectively, accumulated a greater number of coins. Greater beta coefficient values may be seen as reflecting a sensitivity to the expected value (EV) of the overall outcome (net gain or loss). Yet, a wide range of gain-approach and loss- avoid coefficient values, evident in all samples, suggests that motivational conflicts are not necessarily resolved simply through EV calculations [63–64], and that risk aversion and or loss aversion may underlie the avoidance in excess of that predicted by a negative EV. One factor that appears to influence such decision making appears to be trait anxiety [34, 65], as discussed next.

#### Passive avoidance and anxiety

A notable departure from EV-based conflict resolution manifests in the task in relatively lower gain-approach coefficient values (smaller β_Gain_ values). From a psychological-behavioral perspective, this effect reflects *passive avoidance*, i.e., diminished approach behavior in the context of a motivational conflict [14]. Importantly, while Gray originally framed passive avoidance as behavioral inhibition in response to anticipated loss [3], more recent accounts of his theory have expanded this view to include conflict-driven regulation [8], similar to the current task. From this perspective, diminished approach can be adaptive, reflecting a strategy to minimize risk in contexts where pursuing a reward is accompanied by a high potential cost. Such relinquishing of potential rewards due to risk of an aversive outcome is distinguished from *active avoidance*, which involves actively removing oneself from the presence of an imminent threat [27]. These behaviors are overtly different and accordingly rely on differential engagement of dedicated neural circuitry [27, 66], yet the distinction between them is often absent in human research [e.g., 67–69]. The Doors task enables examination of passive avoidance (via magnitude of β_Gain_ coefficients) independently of active avoidance (via magnitude of β_Loss_ coefficients) and thus has utility in mechanistic research on these distinct behaviors.

Clinically, excessive passive avoidance is a key feature of pathological anxiety that is highly impairing and is believed to be major factor in its maintenance [14, 30, 70–72]. Indeed, we found higher levels of trait anxiety to be associated with stronger passive avoidance tendencies among young adults (Studies 1 and 2) and pediatric anxiety patients (Study 4) (but not in the online sample, which evidenced an inverse anxiety-passive avoidance association). These findings are in line with prior empirical work [28, 34, 73], as well as theoretical frameworks of aberrant decision making in motivational conflicts in anxiety [14, 27, 30, 70, 74–76]. Importantly, these findings inform on the potential utility of the Doors task in capturing anxiety-related behavioral effects across some populations, including pediatric anxiety patients [77].

At the same time, it is important to note differences in findings across studies which should be acknowledged when considering their replicability such that it is not overstated. Associations of behavior parameters with anxiety severity were observed using different anxiety assessment tools (e.g., STAI in Study 2 and 3, but not 1; diagnostic interview in Study 4; see Table S4 for summary). The use of consistent tools, when possible, may facilitate comparing findings across studies, while employing within-subject designs could help disentangle cohort-related effects from contextual factors tied to delivery format (e.g., in-person vs. online). Another important difference across the studies is that in-lab administration yielded negative associations between anxiety severity and approach-related tendencies, while a positive association was noted in the online-delivery sample. As such, while all studies evidenced links between anxiety and behavior tendencies, it is important to note that replication of anxiety-passive avoidance associations was limited, and to establish the conditions in which these specific effects emerge. Specifically, the nature of study population (e.g., heterogeneous community sample vs. target population such as students or research-oriented youth) or mode of delivery (e.g., at home vs. inperson, in-lab) may elicit different aspects of anxiety, motivation to monetary rewards, and subsequent relations to task performance [78]. For example, the use of monetary incentives in the online study, unlike course credit in the in-lab versions, may have altered the motivational context, potentially amplifying the salience of reward and punishment cues and thereby shifting how anxiety levels relate to approach and avoidance behavior. Alternatively, lab administration of the task involves in-person interactions with research staff which may add a social-evaluative aspect to it which is absent in remote administration [79]; further, the opportunity cost of completing a specific task among online workers may be higher than in other settings. These differences may potentially impact performance and interact with anxious traits in distinct ways and should therefore be examined in future research.

#### Biases in the subjective experience of losses

Another finding that emerged is that while participants consistently gained significantly more coins than they lost during the game, they tended to overestimate both the frequency and magnitude of losses compared to their actual encounters. The tendency to overestimate loss-related information, often termed ‘loss bias’ or ‘negativity bias’, is well established in psychological research [80–81], and is often examined in psychological-economic, decision-making frameworks such as prospect theory, whereby the disutility of a loss exceeds the utility of an equivalent gain, posing a greater psychological impact [63–64].

In one adult sample, anxiety and depressive symptom severity was found to be associated with the magnitude of subjective bias in experiencing loss, though this link was not found in other samples. Thus, although a loss-related bias is robustly elicited by the task, its relationship to clinical symptomatology remains inconsistent. This variability underscores the need for caution in interpreting symptom-related associations and replication. Nevertheless, the tendency to overestimate negative outcomes is conceptually aligned with cognitive theories of emotional disorders, which emphasize attentional, interpretive, and memory-related biases toward negative information [82–85].

#### Key strengths and limitations

The findings in this report underscore several strengths of the Doors paradigm in research on motivated behaviors. First, mapping of parametrically-varying outcome magnitudes onto a continuous behavioral outcome enables the generation of robust, sensitive, and reliable subject-level indices of behavior tendencies. Such indices may be useful for research on the factors that govern motivated behaviors, both in normative and in maladaptive forms, such as in depression and substance use [19, 86]. Furthermore, the parametric combination of gains and losses in the task design and the internal consistency in the task behavior make it amenable to computational modeling frameworks, opening avenues for quantifying behavioral metrics beyond those explored in the current study (see [29, 87]). Another strength of the task design lies in its adaptability and generalizability, as it has been successfully implemented across different populations, including a clinical pediatric sample, and modes of delivery, including lab- and online-based delivery (see [54–55]; for a specific example of online psychological research examining anxiety-related avoidance, see [87]).

Finally, the Doors task captures costly avoidance, an aspect underexplored in avoidance paradigms in which threat may be altogether avoided without meaningful trade-offs (e.g., via button press to prevent an imminent danger). By embedding potential monetary losses, requiring participants to weigh reward and punishment, the task may model typical real-world avoidance behavior, and particularly passive avoidance, more accurately [88].

At the same time, several limitations of this study warrant consideration. First, despite examining the task in multiple samples including a clinical pediatric sample, the present findings must be replicated in additional clinical samples in order to draw stronger conclusions on pathological anxiety-related effects. Future studies may further extend task findings to additional clinical populations associated with aberrant motivated behavior tendencies, such as in individuals suffering from depression or substance use disorders [19]. Further, the inclusion of neuroimaging and psychophysiological measures may add to the validity of behavioral findings by linking behavior to underlying neurobiological mechanisms. Relatedly, although the present study demonstrates the utility of the Doors task for quantifying behavior in motivational conflict, it is necessary to establish additional aspects of its validity through related task assessing aspects of motivated behavior (e.g., vigor, persistence, and effort during initiation and maintenance of behavior). Moreover, given that some clinical associations with behavioral tendencies emerged significantly, but in inverse patterns, across different samples suggests that while some task effects are very robust, others may be more susceptible to specific characteristics of study population or task delivery.

### Conclusions

In conclusion, the current findings support the hypothesis that potential outcomes systematically shape behavior, as captured by the Doors task. Using this paradigm, outcome magnitude-driven behavioral tendencies can be quantified at the individual-subject level, offering researchers a valuable tool for studying these tendencies in ecologically valid decision-making scenarios. Consistent findings across different populations and modes of delivery indicates its utility for use in research, including in research on maladaptive motivated behaviors in psychopathology, while variability in findings encourages research on potential moderators of outcomes.

## General Method

### Participants and Procedure

To examine the capacity of the Doors task to quantify motivational-behavioral tendencies within conflict and test our hypotheses, we examined a total of 228 participants across 4 studies, representing different populations, including varying ages and psychopathological statuses, and modes of delivery. All participants completed the same task, with minor variations. In Study 1, we tested the capacity of the task to reliably index individual differences in motivated behaviors in a sample of young adults (N=20). In Study 2, we sought to replicate task effects in a larger young adult sample (N=36). In Study 3, we extended the task to an easy-to-disseminate, nonlaboratory, online setting via the Prolific platform (N=120). Finally, in Study 4, we administered the task in a sample composed of pediatric anxiety patients and healthy controls (N=51). Written informed consent/assent was obtained from all adult/youth participants, respectively. Study procedures were approved by the Institutional Review Boards of Reichman University and the National Institute of Mental Health (NIMH). All methods were performed in accordance with relevant guidelines and regulations. Participants received monetary compensation or academic credit for their participation.

### The Doors approach-avoid behavioral task

**Fig. 7.**
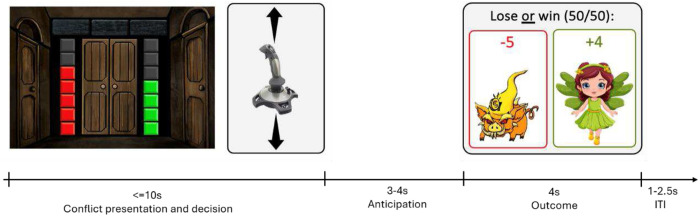
Schematic depiction of the Doors approach-avoidance behavioral task. In each trial, a conflict between potential loss and gain magnitudes is presented (in this case: losing 5 coins or gaining 4 coins). The participant uses a joystick to determine the extent of their desire to engage in that gain vs. loss scenario, with a 10s maximal deliberation time. Behavior is quantified on a continuous 0–100 scale, with greater chosen proximity to the door leading to greater chances of it opening. If the door opens, the outcome is randomly determined and displayed to the participant. ITI = intertrial interval.

We developed the Doors task to independently quantify approach and avoidance behavior tendencies within a motivational conflict. The task was programmed in PsychoPy [89], an open-source platform widely used for cognitive and behavioral experiments. In this task, participants are informed that they would encounter a series of animated doors, behind each of which is either a gain or loss of game coins. The goal is to maximize winnings by accumulating coins, which are converted into money awarded at the end of the task. Each trial (see Fig. 7 for schematic trial time course) features a combination of potential gain (1–7 coins) and loss (1–7 coins); the magnitudes of potential gain and potential loss are represented by the number of green and red bars on each side of the door (side counterbalanced across participants), respectively (see Fig. 7).

When presented with each door, the participant determines the extent to which they choose to engage in that particular gain vs. loss lottery using a standard joystick (Extreme 3D Pro Joystick, Logitech). The joystick movement provides a continuous position output along a 0–100 scale, where 0 represents full avoidance (joystick pulled fully back) and 100 represents full approach (joystick pushed fully forward). The chosen joystick position is linearly mapped onto the probability of the door opening (i.e., whether the door opens or remains closed), ranging from approximately 0% to approximately 100%.

At the start of each trial, the participant is located midway along the distance scale (value of 50). Given a certain combination of green bars and red bars (indicating potential gain and loss magnitudes, respectively), pushing the joystick increasingly forward with increasing potential gain indicates stronger gain-approach behavior, as willingness to engage in the risky outcome situation increases (i.e., leading to higher probability that the door will open). Relatively diminished gain-approach behavior (i.e., not moving as much forward with increasing potential gains, or inaction) therefore reflects passive avoidance - the tendency to forgo potential rewards when the situation also involves possible loss [14]. Pulling the joystick increasingly back with increasing potential loss (i.e., leading to lower probability that the door will open) may reflect active avoidance behavior, as a greater potential loss increasingly leads the participant to actively remove themselves from the possibility of the loss materializing. The door’s relative size on the screen is dynamically increased or decreased corresponding to joystick movement. The participant is instructed to indicate their chosen location by a trigger press, and within 10 seconds (if a participant makes their choice before the full 10 seconds have passed, the task advances immediately). Based on the chosen location, if the door opens (see above), a single outcome— either the gain or loss—is determined randomly with equal probability (50-50 for gain or loss) and represented by either animated coins or a monster, along with the number of coins gained or lost displayed on the screen, respectively; if the door does not open, no outcome is delivered. A corresponding sound is paired with the outcome image. Thus, on each trial, the participant is presented with a conflict involving potential monetary gain or loss and should integrate the potential outcomes to then index the extent to which they wish to engage in the lottery.

The task was administered in 2-3 blocks, separated by short rests, with a duration of approximately 10 minutes/block. Each block consisted of 49 trials comprising all combinations of green bars (1-7 coins gain) and red bars (1-7 coins loss). The trial order was random.

Before the task and after each run, participants completed a series of questions assessing their fatigue, motivation, anxiety, and mood. Prior to starting the task, the experimenter detailed the instructions and confirmed that the participant understood them. Participants also underwent a brief training round to familiarize themselves with the joystick and task. (see details below for some variations). At the beginning of the task, the participant was awarded a number of coins (5-7 coins, randomized), which did not affect the total number of coins earned. At the end of the task, the total number of accumulated coins was converted into a monetary bonus paid in addition to the base participation compensation.

### Data analysis

To quantify subject-level behavior as a function of potential outcome magnitudes, we conducted a linear regression for each individual participant whereby chosen proximity to the door in each trial was predicted by the magnitude of potential gain (reward) and the magnitude of potential loss (punishment). To estimate the effect of conflict level on behavior, a conflict predictor was quantified as the absolute difference between gain and loss magnitudes, subtracted from 7 (such that greater values reflect greater conflict). Additionally, the interaction terms between reward, conflict and punishment were entered, as was the trial’s reaction time (RT). Trials with RT<150 ms were excluded from analysis; trials with RT=10,000 ms (maximal allotted deliberation time) were also excluded from analysis since the participant failed to make a behavioral choice. All entered predictors were mean-centered to diminish collinearity. Analyses focused on the effects of potential gain (*β_Gain_*) and loss (*β_Loss_*) magnitudes, as well as conflict magnitude (*β_Conflict_*), on behavior. The *β* coefficients (*β_Gain_* and *β_Loss_*) reflect the strength of association between the magnitude of potential gain or loss and the participant’s chosen proximity to the door. Within this framework, positive *β_Gain_* coefficients represent the strength of gainapproach tendencies with lower relative values reflecting a tendency for passive avoidance (forgoing rewards within motivational conflicts), while negative *β_Loss_* coefficients (decreasing door proximity with increasing potential loss) reflect active avoidance tendencies [8, 14]; and *β_Conflict_* coefficients correspond to conflict sensitivity, with positive values reflecting approach tendencies with increasing conflict levels, and negative values reflecting conflict avoidance [14, 90].

The primary analysis in each study examined whether *β_Gain_*, *β_Loss_* and *β_Conflict_* can meaningfully predict behavior within motivational conflicts. This was tested within a single linear mixed-effects model (using *lme* from the *nlme* R package v3.1-165 [91]) that predicted trial-level chosen distance from the door based on the aforementioned predictors, as well as age and sex, with participant as a random effect. T-tests (vs. 0) examined the significance of predictors of interest. Additionally, reliability of indices across task runs was assessed using intra-class correlation coefficients (ICC3, average of fixed raters; ICC, *psych* R package). In all studies, variables with skewness of >|1| were log-transformed to increase distribution symmetry, followed by winsorizing of outliers (using *Winsorize* default settings, *DescTools* R package), prior to calculating Pearson correlations to examine associations between behavior predictors and different symptoms or traits. Analyses and plots were conducted in R [92]. All statistical tests used significance level of 0.05 (two-sided).

#### Study 1

##### Participants

A total of 20 young adults (15 females; *M* age = 24.50 years, *SD* = 1.70, age range = 22–28) took part in the study for academic credit and payment at Reichman University. Participants did not receive any bonus payment at the end of the experiment.

##### Measures

As noted above, we extracted the *β_Gain_*, *β_Loss_* and *β_Conflict_* motivated behavior indices at the individual-subject level (see General Method).

In addition, we assessed trait anxiety and depression symptom severity. Anxiety symptom severity was assessed using two standard questionnaires. The trait subscale of the State-Trait Anxiety Inventory (STAI) is a valid and reliable instrument which consists of 20 items pertaining to anxiety-related symptoms rated on a 4-point Likert scale (1=“not at all”, 4=“very much so”) [93]. The total score was used to index anxiety severity. Mean total STAI score in the sample was 38.80 (*SD* = 13.38) and Cronbach’s alpha = 0.93. The Generalized Anxiety Disorder-7 (GAD-7) is a valid and reliable questionnaire that likewise assesses severity of anxiety symptom through seven items scored on a 4-point Likert scale (0=“ not at all”, 3=“ nearly every day”) [94]. Mean total GAD-7 score in the sample was 5.60 (*SD* = 5.89) and Cronbach’s alpha = 0.96.

We assessed depressive symptom severity using the PHQ-9 (Patient Health Questionnaire-9), a valid and reliable instrument consisting of 9 items indexing depression-related symptoms rated on a 4-point Likert scale (0=“not at all”, 3=“nearly every day”) [95]. The total score was used to index symptom severity. Mean total PHQ-9 score in the sample was 5.70 (*SD* = 6.64) and Cronbach’s alpha = 0.95.

#### Study 2

##### Participants

A total of 37 young adults (28 females, *M* age = 25.14 years, *SD* = 3.65, age range = 18–35) who were undergraduate students took part in the study for academic credit and payment at Reichman University. Participants received a bonus payment comparable to approximately $3.00–$3.50 USD at the end of the experiment in addition to their base participation compensation.

##### Anxiety and depression symptoms

Similarly to Study 1, anxiety symptom severity was assessed using the STAI, *M* = 39.86 (*SD* = 10.59) and Cronbach’s alpha = 0.91; unlike Study 1, this study did not employ the GAD-7 scale. Depressive symptoms severity was assessed using the PHQ-9, *M* = 6.11, (*SD* = 5.22) and Cronbach’s alpha = 0.86.

##### Task

Study 2 utilized the same task, with a few modifications. First, only two runs were conducted (98 trials total) instead of three, to examine whether effects are observed with fewer trials. Second, trial-level gain outcome presentation now included a fairy figure in addition to the number of coins won (to balance the presentation of a monster figure and coins lost in loss trials). Finally, after the task ended, participants were asked to subjectively evaluate their experience of gains and losses by answering the following questions on a 0–10 scale: “How often did you win coins (see a fairy) when the door was opened?” (gain frequency, rated *never* to *always*); and “How often did you see a monster when the door was opened?” (loss frequency, rated *never* to *always*); “How many coins do you think you won?” (gain magnitude, rated *few* to *many*); and “How many coins do you think you lost?” (loss magnitude, rated *few* to *many*). Due to technical issues, data from these questions were unavailable for four participants.

#### Study 3

##### Participants

A total of 120 adults (60 females) featuring a broad age range (*M* age = 37.91 years, *SD* = 12.25, age range = 21–79) were recruited through Prolific platform (https://www.prolific.com/) and the Pavlovia website (Open Science Tools, https://pavlovia.org/). We recruited participants who are age 18 and above, fluent in English, located in the US, and have approval rate at of least 95%, indicating reliable task performance. Participants were compensated $20 for participation. In the task, they received $10 as endownment, and won or lost additional $0.10 for each gained or lost coin, respectively. At the end of the task, they received the final money as bonus (*M* bonus=$14.5, *SD*=$4.8, range=$0.2-$27.1). Written informed consent was acquired online from participants.

Instead of a joystick, participants determined their locations using keyboard presses (clicks on the left-arrow and right-arrow keys moved the participant closer or away from the door, respectively). Given that online task performance via Prolific is more difficult to control than in lab settings, we excluded participants for whom trials with the maximal RT (10,000 ms) comprised the vast majority of trials (i.e., participants with ≥80% of trials with maximal RT), suggesting they did not follow task instructions meaningfully as they did not choose locations in time. This led to the removal of 36 participants, and a final sample of 84 participants on which analyses were performed. Prior research using online platforms, such as Prolific and MTurk, has reported similar rates of exclusion due to inattentive or noncompliant responding [96–98]. Of note, retaining all participants by not using this exclusion rule led to similar results (see Supplement); we chose to focus here on participants who we assess to have performed the task more meaningfully. Before starting the task, detailed instructions were presented on the screen, and participants completed a brief practice round to familiarize themselves with the task controls.

##### Anxiety and depression symptoms

Anxiety symptom severity was assessed using the trait subscale of the STAI; *M* = 42.89 (*SD* = 12.21) and Cronbach’s alpha = 0.94. Additionally, the GAD-7 (*M* = 42.89, *SD* = 12.21; Cronbach’s alpha = 0.92) and PHQ-9 (*M* = 42.89, *SD* = 12.21; Cronbach’s alpha = 0.90) scales were administered; findings with these scales are reported in the Supplement.

#### Study 4

##### Participants and procedure

A total of 51 youths, including 27 treatment-seeking, medication-free patients with pediatric anxiety disorders (ANX; 12 females, *M* age = 13.42 years, *SD* = 3.17, age range = 8–18) and 24 healthy volunteers (HV; 12 females, *M* age = 13.28 years, *SD* = 3.14, age range = 8–18) took part in this in-lab study at the NIMH. A 3-run variant of the task was completed in a visit following clinical assessment (see below). Following Study 3, we excluded 3 participants (1 HV, 2 ANX) with non-significant *β_Gain_* and *β_Loss_* coefficients or insufficient trials (<49; see Supplement), and another one for technical reasons, leaving a final sample of N=47. The groups did not differ in age, *p*=0.87, or biological sex, *p*=0.67. Participants received a bonus payment of approximately $10 at the end of the experiment.

##### Diagnosis and anxiety symptom severity

Full details about the diagnostic process are provided in the Supplement. Briefly, all participants were interviewed by trained clinicians using the Kiddie Schedule for Affective Disorders and Schizophrenia for School-Age Children-Present and Lifetime Version (KSADS-PL; [99]). Patients met criteria for generalized anxiety, social anxiety, and/or separation anxiety disorder as their primary source of distress. Healthy participants were required to be free of any diagnoses.

Given the presence of two diagnostic groups (ANX, HV), we compared them in terms of task indices using analysis of variance (ANOVA). In secondary analysis, we examined dimensional associations between anxiety symptom severity and task measures, for comparability with the previous studies. Anxiety symptom severity was assessed using the child- and parent-report versions of the Screen for Child Anxiety Related Emotional Disorders (SCARED), a frequently-used, psychometrically-robust instrument for assessing pediatric anxiety symptoms [100–101]. The SCARED includes 41 items pertaining to anxiety-related symptoms or behaviors; each item is rated on a 3-point Likert-type scale (0 = not true, 2 = very true). As in prior research [102], the average of child- and parent-reported SCARED total scores was used in analyses to reduce reporter discrepancies [103]. The Cronbach’s alpha for the SCARED scores was 0.96 for both the child and parent reports. The ANX group had significantly higher total scores than the HV group, *t*(49)=8.88, *p*<0.001.

## Data Availability

Data, analysis code, and study materials (including task scripts and stimuli) used in this research are publicly available on the Open Science Framework (OSF) at https://osf.io/asyfq/ [104]. Please note that data from the final sample cannot be shared publicly due to NIH policy restrictions.
